# Exploring the Potential of Fungal Biomass for Bisphenol A Removal in Aquatic Environments

**DOI:** 10.3390/ijms252111388

**Published:** 2024-10-23

**Authors:** Kamila Wlizło, Marek Siwulski, Beata Kowalska-Krochmal, Adrian Wiater

**Affiliations:** 1 Department of Industrial and Environmental Microbiology, Faculty of Biology and Biotechnology, Maria Curie-Skłodowska University, Akademicka 19, 20-033 Lublin, Poland; adrian.wiater@mail.umcs.pl; 2 Department of Vegetable Crops, Faculty of Agriculture, Horticulture and Biotechnology, Poznań University of Life Sciences, Dąbrowskiego 159, 60-594 Poznań, Poland; marek.siwulski@up.poznan.pl; 3 Department of Pharmaceutical Microbiology and Parasitology, Faculty of Pharmacy, Medical University of Silesian Piasts in Wroclaw, Borowska 211a, 50-556 Wroclaw, Poland; beata.kowalska-krochmal@umw.edu.pl

**Keywords:** water treatment, higher fungi, mushrooms, xenobiotics, sorption

## Abstract

Bisphenol A is a plastic component, which shows endocrine activity that is detrimental to humans and aquatic ecosystems. The elimination of BPA from the environment is one of the solutions for BPA contaminant management. Adsorption is a cost-effective, easy-to-use method generating low harmful byproducts; nevertheless, contaminant sorbent treatment is a challenge that still needs to be addressed. Fungal fruiting bodies biomass is rarely studied sorbent but is promising due to its high polysaccharide content and availability. Our preliminary studies showed BPA sorption (100 mg/L) by 50 cultivated and wild fungi. The cultivated species: *Clitocybe maxima* (82%), *Pholiota nameko* (77%), and *Pleurotus columbinus* (74%), and wild fungi *Cantharellus cibarius* (75%) and *Lactarius deliciosus* (72%) were the most efficient. The biomass was able to sorb BPA over a broad range of temperature and pH levels, with an optimum at 20 °C and pH 7. Although saturation of sorbents was rapid, the regeneration process using ethanol was effective and allowed to recover up to 75% of sorbents’ initial efficiency. A single use of 1 g of sorbent would allow the treatment of 8.86 to 10.1 m^3^ of wastewater effluent, 16.5 to 18.7 m^3^ of surface water, and 411 to 469 m^3^ of drinking water, assuming the concentrations of BPA reported in the literature.

## 1. Introduction

Bisphenol A (BPA) is commonly used in the production of polycarbonate plastic and epoxy resin, and as a result, it can be found in a wide range of everyday items, including plastic food containers, coatings of beverages and food cans, plastic toys, and infant feeding bottles. Moreover, BPA is a key ingredient in paint, thermal paper, compact disks, building materials, dental sealants, and more. Unfortunately, BPA can be released from products and enter the body through ingestion, inhalation, dermal adsorption, and even maternofetal transmission [1,2,3]. Its chemical structure, which includes two benzene rings and two -OH groups, enables BPA to bind to the estrogen receptor (ER) [4,5]. This can have significant implications for living organisms, including negative effects on human health and ecosystem balance. In humans, BPA has been linked to interference with the reproductive system leading to issues such as infertility, abnormal sperm production, feminization of the genitals in males, as well as ovulation problems and decreased oocyte quality in females. It can also cause fetal malformations during pregnancy [3,6,7,8]. Some studies have also suggested that BPA may contribute to the development of certain diseases, such as cancer and diabetes. In the realm of animals, the most detrimental effects in aquatic species are observed at the base of the food chain, specifically in *Daphnia magna*, where BPA is highly toxic. The toxicity of BPA has also been documented in smaller fish, such as medaka (*Oryzias latipes*), while in larger species, exposure to BPA leads to feminization of males and impairment of the reproductive system, resulting in a reduction in population size in the environment. BPA is present persistently in the environment. It is found in soil and groundwater as a result of leakage from plastic waste in landfills, and it is introduced to wastewater from factories during the production process. However, its low treatment efficiency causes it to contaminate the water and activated sludge, which is released into the natural environment [9,10,11]. It is estimated that the global consumption of BPA will reach 10.6 million metric tons by 2022, a figure that underscores the magnitude of the issue at hand [12]. To mitigate the negative impact of BPA on the environment, regulations have been put in place to restrict its release. However, despite these measures, numerous products still contain BPA, and the accumulation of residual waste continues to contaminate the environment. Treatment plants are unable to effectively remove BPA, highlighting the need for the development of more efficient purification methods. Adsorption, along with other cost-effective methods such as membrane techniques, advanced oxidation processes, and constructed wetland systems, is frequently cited in the literature as a low-cost, user-friendly process that generates minimal harmful byproducts. Despite some drawbacks, such as the treatment of desorption waste liquid and the desorption process itself, which can sometimes be problematic, adsorption remains a widely studied method [13,14,15]. Researchers are primarily focusing on the development of effective sorbents that are easily regenerated and economically viable to produce and utilize. In a review paper published by Bhatnagar and Anastopoulos, various adsorbents made from natural materials such as clay, zeolite, and activated carbon synthesized from agricultural waste, as well as synthetic polymers, nanomaterials, and even graphene-based materials, were discussed regarding their ability to remove BPA [16]. However, no sorbents based on Basidiomycota fruiting bodies biomass were mentioned. While chitosan, a fungal polymer, has been used in similar studies, it is often modified through the addition of functional groups or combined with other polymers. Given that the fungal cell wall is composed of polysaccharides up to 90%, the promising results obtained with chitosan prompted further research into other cell wall polysaccharides, such as α- and β-glucans [17]. Fungal mycelium was tested as a promising candidate for the adsorption of pollutants such as BPA due to its high surface-to volume ratio, which has been demonstrated in studies on mycelium of Ascomycota and certain Basidiomycota species, such as *Trametes versicolor* [18,19]. However, these experiments were conducted using mycelium specially produced for enzymatic degradation. The use of fruiting biomass for adsorption, on the other hand, bypasses conditions that are unfavorable for enzyme action, and given the scale of mushroom production, this is the great source of biomass including waste biomass from cultivation that has not been introduced into the food market. This aligns with the principles of a circular economy [20]. Furthermore, BPA treatment via adsorption utilizing fungal biomass as a sorbent warrants consideration, as this method does not generate intermediate or final toxic products, in contrast to degradation methods employing, for instance, lignin modifying enzymes (LME) or advanced oxidation processes.

The purpose of this research was to assess the biomass of fungal fruiting bodies during a screening test for BPA removal. Additionally, the study aimed to investigate the process conditions, the potential for regeneration, and reuse of the most effective biomass with predictable yield per cubic meter of water treated.

## 2. Results and Discussion

### 2.1. Screening Test

Biosorption of BPA has been researched utilizing a variety of sorbents, including magnetic composite materials, clays, and chitosan, as well as advanced materials, such as carbon nanotubes and graphene-based materials. More recently, agricultural waste materials have gained popularity as sources of activated carbon for use as a sorbent. This approach not only addresses the issue of accumulated waste but also helps to reduce energy expenditure in producing new sorbents. Moreover, this strategy aligns with the principles of a circular economy and the European Union’s “green deal” regulations [10,20]. The utilization of mushroom waste as a source of sorbent material is also gaining traction, due to the significant global production of mushrooms (44 million tons in 2023) and the potential for a rich source of biomass from post-harvested waste and outdated edible parts [21]. Although mushroom and Basidiomycota fungi have rarely been studied for their biosorption capabilities, the biosorption of xenobiotics, including BPA, by fungal biomass has been considered as an additional mechanism accompanying their enzymatic degradation by live mycelia. As a standalone mechanism, the biosorption capabilities of inactivated mycelium have rarely been studied [22,23].

**Figure 1:**
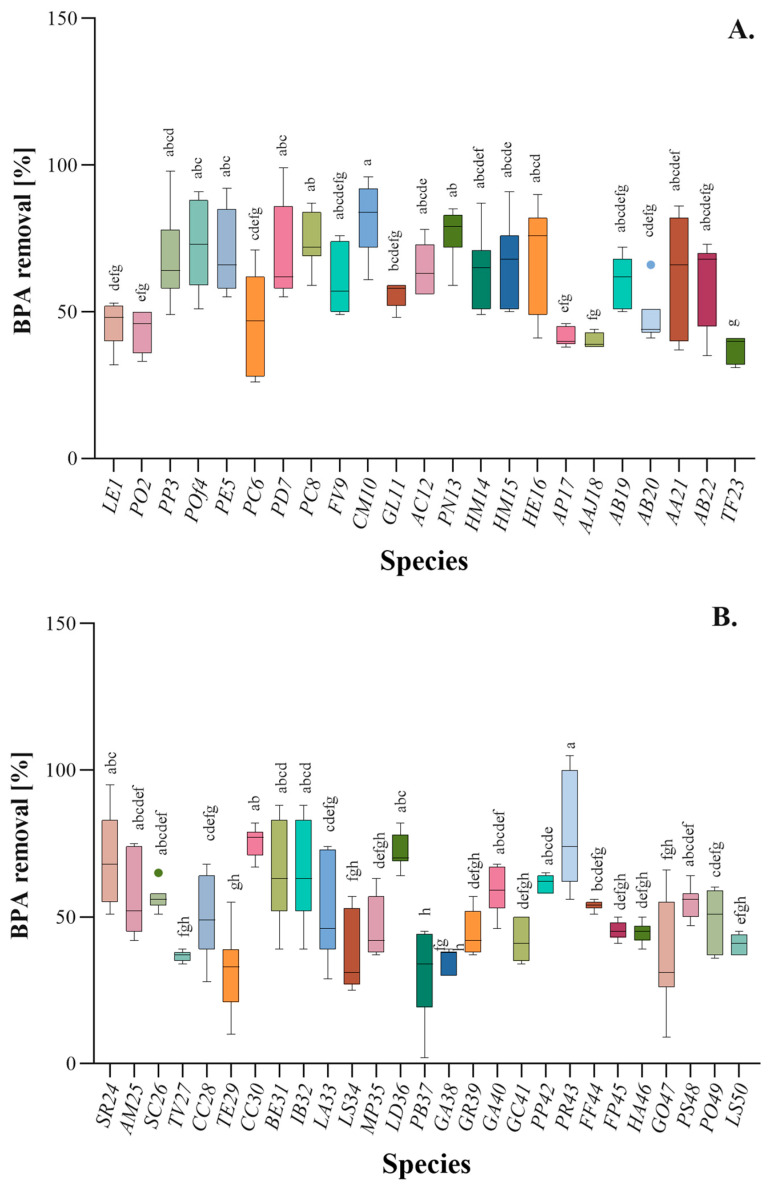
Results of the BPA removal by homogenates of cultivated (**A**) and wild (**B**) fungi. One-way ANOVA with Tukey’s post-hoc test, *p* < 0.05. Bars with different letters (a–h) are statistically significantly different from each other (*n* = 9); dots indicate the most divergent results.

Our investigation commenced with a screening test aimed at identifying a group of fungi capable of effectively removing BPA from aqueous environments using their fruiting body biomass. The concentration of BPA tested was 100 mg/L, which was essential for detection given the method employed, including the measurement of sample background absorbance at 275 nm. A similar method with the same concentration was used by Rovani et al. for BPA adsorption on silica nanoparticles [24]. However, this method proved sufficient for screening purposes. The homogenates of fifty fungal fruiting bodies were categorised into two types, namely cultivated (A) and wild (B) fungi, and were subsequently incubated with a BPA solution. The level of residual BPA in the supernatant was evaluated, and the findings are depicted in Figure 1A,B. The elimination of BPA in Group A was marginally greater than in Group B, with an average elimination of BPA in Group A being 60% ± 12 compared to Group B at 51% ± 13. Therefore, the most efficient BPA-removing fungi from both groups were selected for further study.

The selected fungi in Group A were *Clitocybe maxima* (82%), *Pholiota nameko* (77%), and *Pleurotus columbinus* (74%). Notably, four subsequent and comparably efficient fungi in this group belong to the *Pleurotus* genus. This is a positive phenomenon because of wide-spread cultivation of *Pleurotus* species generating high amount of waste biomass (stems) that is not introduced into the food market [21]. Our findings can be compared to studies on the removal of BPA using inactivated mycelium of *Pleurotus eryngii*, which was immobilized on Amberlite XAD-4., which removed 90% of BPA at a concentration of 120 mg/L, but the influence of Amberlite on the outcome cannot be excluded [25].

The most efficient fungi in Group B were *Phellinus robustus* (77%) and *Cantharellus cibarius* (75%). However, due to the large standard deviation of the results obtained for *Phellinus robustus* and the difficulties in processing its fruiting body biomass, it was excluded from further study, and *Lactarius deliciosus* (72%) was selected instead. This group included wild fungi and one of them, *Trametes versicolor*, was also tested in terms of BPA adsorption by Nguyen and colleagues [19]. In their study, the efficiency reached 30% of BPA elimination from a 50 µg/L solution by inactivated whole-cell mycelium, while in our study, it was approximately 35% of BPA removal. The capacity of fungi to sorb substances is largely attributed to the content of oligo and polysaccharides in their cell walls (CW), which provide a significant number of binding sites. The structure of fruiting bodies, which differs from one fungus to another, may also affect the efficiency of sorption [26].

Chitin content, as well as hydrophobins, are important factors that influence the water sorption capacity and hydrophobic characteristics of the cell wall, which in turn impact the ability of fungi to bind xenobiotics [27]. It is worth noting that in our study, certain types of fungi with gelatinous or cotton-like structures, such as *Auricularia auricula-judae* and *Ganoderma applanatum*, respectively, showed relatively low BPA sorption efficiencies.

### 2.2. Time of Incubation

**Figure 2:**
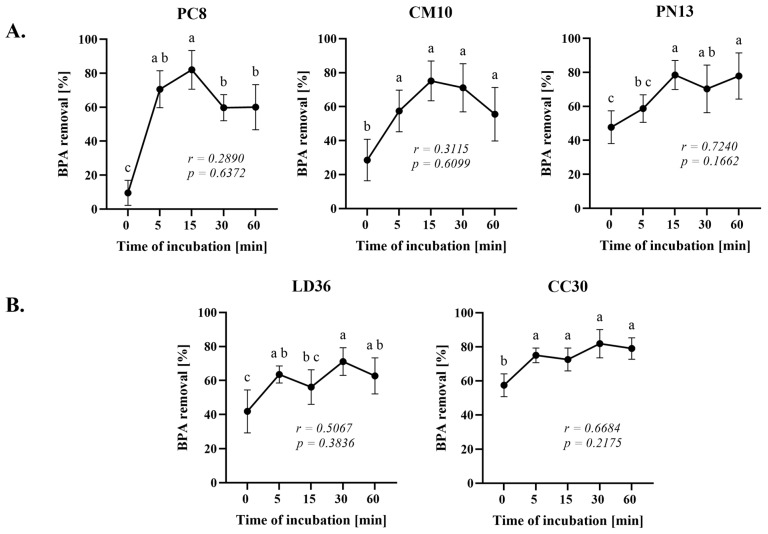
Results of incubation time on the rate of the BPA removal by homogenates of cultivated fungi (**A**) and wild fungi (**B**). One-way ANOVA with Tukey’s post-hoc test, *p* < 0.05. Bars with different letters (a–c) are statistically significantly different from each other (*n* = 9). The correlations were analyzed using Pearson’s correlation coefficient *r* and the statistical significance *p* of this coefficient.

The optimal sorbent should exhibit high effectiveness and expedient BPA extraction within a brief period. Hence, the initial stage of evaluating the conditions for BPA removal commenced with establishing an appropriate reaction time. The homogenates of selected, the most efficient fungi were exposed to BPA solution for varying lengths of time, ranging from 0 to 60 min at room temperature and the outcomes were depicted in Figure 2. In both sets of results, typical trends were evident. In Group A, the most efficient outcomes were obtained after 15 min of incubation, while in Group B it was 30 min.

In both groups, the binding of BPA to homogenates occurred relatively swiftly in PN13, CC30, and LD36 fungi, with 61, 78, and 60% of the maximum efficiency, respectively, at time 0. A brief contact duration between the sorbent and sorbate, coupled with high efficiency, is advantageous as it allows for more water to be treated in a shorter amount of time. A rapid increase of BPA removal is typically observed in adsorption studies followed by reaching a maximum level and remaining constant [28,29]. Our results, however, demonstrated superiority over other tested adsorbents. The efficiency of BPA elimination by activated carbon AC-40 was significantly lower, reaching only 22% of its maximum efficiency after 30 min of incubation, or 24% of maximum efficiency in BPA removal by CO_2_-activated tire pyrolysis char after 5 min of incubation. Comparable results were only obtained using the KOH-activated variant of tire char and both synthesized and commercial chitosan, with efficiencies reaching approximately 50% of their maximum potential. On the other hand, in our experiment, extending the incubation period over 30 or 60 min, resulted in a decrease of adsorption, which was also observed in several studies [30,31,32]. This result suggests a shifting of equilibrium towards desorption of BPA from the sorbent, which was particularly clear for our PC8 and CM10 species and suggests additional experiment for desorption kinetics in the future studies.

The Person’s correlation test showed a high correlation between the time of incubation and the percentage of BPA removal for the CC30 and LD36 fungi, and a very high correlation for the PN13 species, although no correlations were statistically significant. As a result of this experiment, 15 min of incubation for fungi in Group A and 30 min for fungi in Group B were selected as the optimum.

### 2.3. Influence of Temeprature

**Figure 3:**
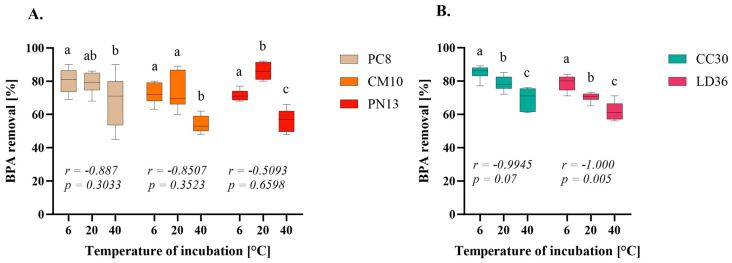
Results of incubation temperature on the rate of the BPA removal by homogenates of cultivated fungi (**A**) and wild fungi (**B**). One-way ANOVA with Tukey’s post-hoc test, *p* < 0.05. Bars with different letters (a–c) are statistically significantly different from each other (*n* = 9). The correlations were analyzed using Pearson’s correlation coefficient *r* and the statistical significance *p* of this coefficient.

Evaluating the impact of temperature on wastewater treatment is an important aspect of new sorbent research that is often overlooked. Consequently, an investigation was conducted to assess the feasibility of BPA removal at various temperatures, commencing with 20 °C, which is considered representative of the temperature of wastewater that is typically found within the range of 10–20 °C during most of the year [33]. In addition, two extreme temperatures of 6 and 40 °C were also tested to account for potential fluctuations beyond the typical range [34]. The time of incubation was selected for each species based on the results from the previous experiment. As depicted in Figure 3, the lowest BPA removal efficiency was observed at 40 °C for all tested fungi. However, the results for fungi in Group A were not clearly differentiated between 6 and 20 °C. The results for species PN13 were significantly higher at 20 °C compared to the other temperatures. In contrast, the results for Group B showed higher efficiency at 6 °C, which decreased with increasing temperature. The correlation between temperature and BPA removal efficiency was high for fungi CC30 and LD36 and statistically significant for the LD36 strain in the Pearson’s correlation test. One of the potential explanations for such correlation may be the influence of temperature on the solubility of the substance and consequently on the adsorption rate. The underlying principle posits that lower temperature reduces a substance’s solubility and promotes higher adsorption. This could theoretically result in a 100% BPA elimination rate at 6 °C. Indeed, the elimination rate is marginally higher at 6 °C compared to results obtained at 20 °C for CC30 and LD36 species, but it did not reach 100%. This observation suggests substantial BPA solubility even at lower temperatures. Furthermore, comparison of the absorbance of control BPA solutions incubated at tested temperatures and centrifuged revealed no discernible difference, confirming that BPA did not precipitate. Interestingly, more efficient BPA and its analogues removal at lower temperatures was also observed in treatment plants [11]. Additionally, it is plausible that the high dose of BPA interfered with observations. A concentration of 100 mg/L BPA may have saturated all binding sites, preventing 100% binding of the substance. Application of a lower dose of BPA would potentially allow binding of all its molecules and yield more definitive results.

The correlation between BPA adsorption efficiency and temperature remains unclear. In studies conducted on free and immobilized carboxymethyl cellulose-lignin composite beads, the fungal biomass of *Trametes trogii* demonstrated an increase in BPA adsorption efficiency with rising temperature [35]. Similar results were reported by Heo and colleagues (2019) for bamboo biochar (BC) and its supported magnetic CuZnFe_2_O_4_ variant (CZF-BC) [36]. However, studies conducted on other types of BC made from grapefruit peels showed a correlation in which higher temperatures led to a decrease in BPA adsorption [37]. This is consistent with our findings, although the decrease in efficiency of grapefruit BC was more pronounced than ours. The broad range of BPA sorption by fungal fruiting bodies biomass offers several advantages over other temperature-dependent biotechnological methods or may be useful in combination with them for wastewater treatment. This may help address the challenge of effective BPA elimination at lower temperatures, as the activity of microbiological wastewater treatment (BWT) or enzymatic degradation by lignin-modifying enzymes (LME) is limited, with optimal temperatures starting at 20–25 °C [34,38].

### 2.4. The pH Value Influence

**Figure 4:**
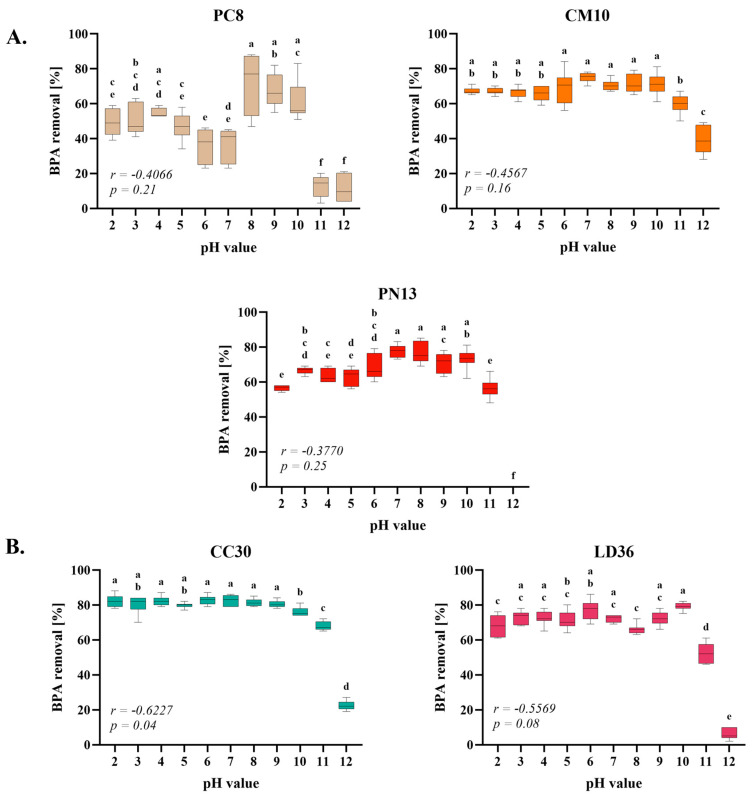
Results of incubation buffer pH value on the rate of the BPA removal by homogenates of cultivated fungi (**A**) and wild fungi (**B**). One-way ANOVA with Tukey’s post-hoc test, *p* < 0.05. Bars with different letters (a–f) are statistically significantly different from each other (*n* = 9). The correlations were analyzed using Pearson’s correlation coefficient *r* and the statistical significance *p* of this coefficient.

In an experiment designed to assess the effectiveness of BPA removal under various pH conditions, the pH range tested was between 2 and 12, utilizing the BR buffer environment. The research findings indicated a broad range of pH values in which the elimination of BPA was feasible (Figure 4). There was no significant correlation between the pH value and the removal of BPA by four tested species as determined by Person’s correlation test. The CC30 species was the only one in which a correlation was observed, with a consistent removal rate of 80% BPA at each pH value, beginning to decrease at pH 10. In contrast, the PC8 species demonstrated lower efficiency at both acidic and highly alkaline pH levels, achieving the highest results between pH 8 and 10. Nevertheless, the BPA elimination efficiency appears to maintain greater consistency at acidic pH values compared to more alkaline conditions. Multiple studies report superior results for BPA adsorption in acidic pH; consequently, it may be postulated that even at pH levels below 2, a certain degree of efficiency will be maintained [36]. These discrepancies in outcomes between species and specific pH values within some species may be attributed to variations in their fungal cell wall (CW) structure and properties. As a complex structure, fungal CW comprises numerous molecules, including α- and β-glucans, aminopolysaccharides, lipids, uronic acids, proteins, hydrophobins, and melanines. Many of these molecules possess positively or negatively charged groups, which influence CW properties. The fungal cell wall may function as both anion- and cation-exchanger due to the presence of amino groups with pKa values of 3.5–4.0 and other groups with pKa values of 8.5–9.0. When placed in an environment with varying pH, the CW adjusts its properties, broadening the range of its action [27]. The molecule of BPA is also influenced by the pH level, as it has a pKa value ranging from 9.6 to 10.2. When the pH value exceeds 9.6, the BPA molecule gains a negative charge on its hydroxyl group, and further exceeding the pH value beyond 10.2 results in a negative charge on the second hydroxyl group, while in acidic pH BPA molecule is protonated [36,39]. The pH has an impact on the ultimate elimination of BPA from the solution, as it influences both the sorbent and sorbate. This was demonstrated in our investigation.

For each case, the efficiency of the process decreased at pH 10–11 and continued to decrease or became 0% at pH 12. However, adsorption was still possible at a highly alkaline pH of 12 for some species, such as CM10 (40% efficiency) or CC30 (20% efficiency). The primary cause of this decrease is most likely the negative charge of the CW surface, which repels the negatively charged BPA molecules. However, despite the maintained sorption efficiency at pH 12 for CM10 and CC30 strains, other mechanisms are probably at play in this process, such as hydrophobic, van der Waals, and hydrogen bonding interactions, as reported by Lee et al. [40]. The stability of sorption across a broad pH range extending to higher alkaline conditions appears to be characteristic of sorbents of natural origin. Results comparable to those obtained in our research were presented in studies utilizing silica nanoparticles derived from sugarcane waste ash, which demonstrated 50% capacity at pH 12, and in investigations on BPA removal employing modified chitin and starch-based resin, wherein high stability of the process was observed up to pH 11 [41,42]. Other sorbents such as porphyrinic porous organic polymer, nano-titanium dioxide, and bentonite showed stability for BPA adsorption up to a pH of 8–9 [40,43,44,45,46]. However, in our research, we observed a stability of approximately 80% BPA elimination up to pH 10. This broad range of effectiveness is favourable, as it aligns with the pH 6.5–9 value of the wastewater [47].

### 2.5. Reuse of Sorbent

#### 2.5.1. Sorbent Stability

**Figure 5:**
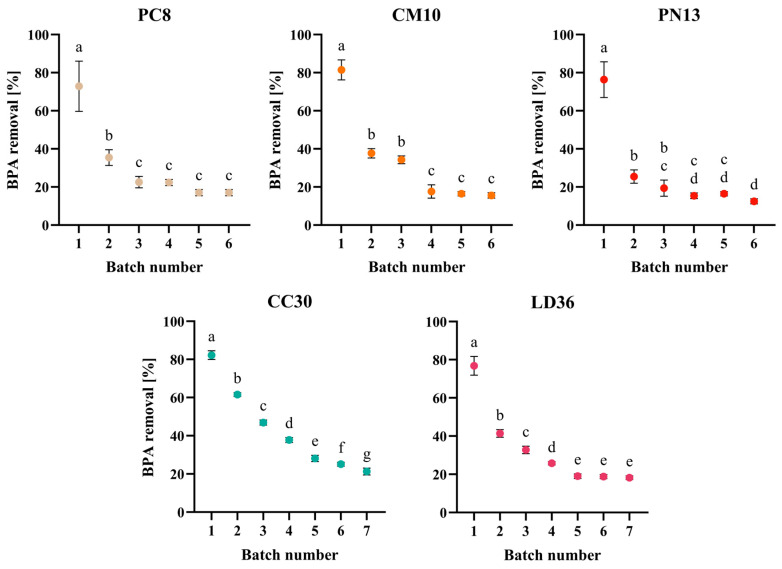
The operational stability of biomass homogenates of cultivated fungi and wild fungi in repeated BPA removal. One-way ANOVA with Tukey’s post-hoc test, *p* < 0.05. Bars with different letters (a–g) are statistically significantly different from each other (*n* = 9).

The reusability of carriers plays a significant role in influencing the overall efficiency of the treatment process. Consequently, a single sample of each homogenate was repeatedly used until all available binding sites were filled. The main objective of the experiment was to determine whether regeneration of the sorbent was necessary immediately following the first use. The biomass utilized in subsequent batches without any cleaning displayed a decrease in efficiency. For the four species evaluated, there was a decrease of approximately 50% between the first and second batch. The filling of the binding sites became slower, and the experiment was continued until only 20–25% of the initial efficiency remained. The saturation progressed slightly more quickly for the biomass of cultivated fungi (six batches) compared to the wild fungi (seven batches) (Figure 5). Notably, the species *Cantharellus cibarius* (CC30) exhibited a higher degree of regularity and slower performance. The biomass of CC30 showed a gradual decline in efficiency, with an average loss of 25% from one batch to the next. Nonetheless, the results obtained indicate that the capacity of sorbents is limited. This rapid capacity decrease is attributed to the high concentration of adsorbed BPA, which rapidly saturates the binding sites.

Nevertheless, it is noteworthy that in certain species, the decrease is rapid in the initial batch and relatively constant in subsequent batches, whereas in other species, the decrease is more gradual. Two potential mechanisms are currently hypothesized: (1) a greater number of free binding sites in species CC30 and LD36, which is observed as their gradual saturation, and/or (2) a higher proportion of desorption in PC8, CM10, and PN13 species, observed as lower BPA elimination. This aligns with the results obtained in the experiment with incubation, wherein the period for efficient BPA elimination was shorter and followed by a decrease in results during further incubation. The possibility might be corroborated during a desorption kinetic study; nevertheless, in both cases, regeneration of the sorbent immediately following the first batch is necessary for further sorbent reusability. The evaluation of unregenerated sorbent is not commonly encountered in many studies, while regeneration between each batch is typical and is generally reported for 5–6 adsorption/desorption cycles [48,49].

#### 2.5.2. Sorbent Regeneration, Reuse, and Estimated Utility

**Figure 6:**
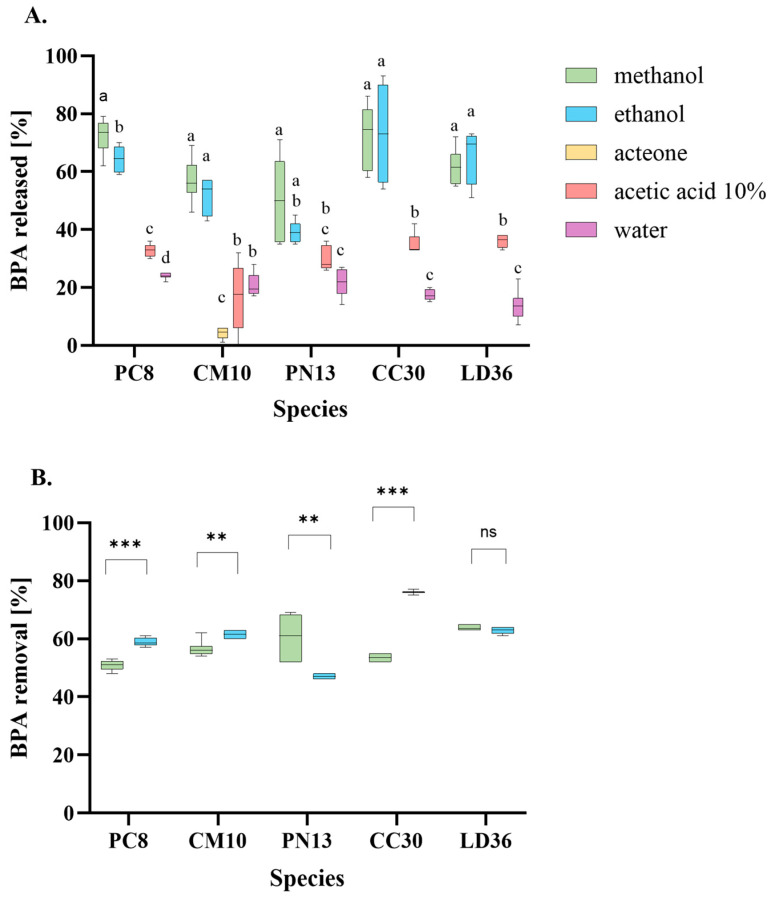
The desorption of BPA bound to biomass homogenates of tested fungi (**A**) and the reuse of fungal biomass (**B**) in BPA removal repeated after desorption. (**A**) One-way ANOVA with Tukey’s post-hoc test, *p* < 0.05. Bars with different letters (a–d) are statistically significantly different from each other (*n* = 9). (**B**) Unpaired *t*-test (*n* = 9), ns—no significance, **—*p* < 0.01, ***—*p* <0.001.

The only product after BPA treatment is contaminated biomass, which requires BPA desorption and its further treatment to reuse. Given that sorbent regeneration is necessary, it is crucial to select an appropriate regeneration solvent. As BPA sorption on fungal fruiting bodies biomasses probably occur through electrostatic interactions, altering the charge would be a feasible approach. One possible solution would be to adjust the pH of the environment; however, since our sorbents exhibited activity over a wide range, only pH 12 can be considered. Nevertheless, using an alkaline solvent may also result in the removal of the alkaline fraction of glucans present in the fungal CW, potentially diminishing the sorbent’s efficiency [50]. An alternative approach is to increase the ionic strength of the solution using sodium chloride or calcium chloride. The use of high concentrations of salt ions may potentially compete with BPA for the binding site, although the exact influence of this factor is not entirely clear and may depend on the nature of the sorbent [16]. Another method may involve a drastic change in pH value. Given the limitation of BPA adsorption in alkaline pH, desorption in NaOH appears to be a rational approach. It has been demonstrated to be an effective desorbate for chitosan/gelatin beads and functionalized cellulose, maintaining good reusability of these sorbents [51,52]. Although effective, high salination or pH value presents an obstacle to the subsequent processing of the desorbate obtained. Thus, the use of organic and inorganic chromatographic solvents for regeneration appears to be a more practical approach. Therefore, this study utilized different solvents with varying elution strengths for BPA desorption. Methanol and ethanol (Figure 6A) were successful in desorbing BPA, with consistent results across all tested fungi, and the CC30 species biomass was the most susceptible to desorption.

Partial desorption was also possible using acetic acid 10% and distilled water, with lower values observed for the latter. Acetone was the only solvent tested that showed no desorption of biomass from the fungi PC8, PN13, CC30, or LD36. Only in CM10 was 10% desorption possible using acetone, and water was a more efficient desorption solution than acetic acid.

Since methanol and ethanol showed equally efficient desorption, a second batch of BPA removal was performed on both regenerated samples. As depicted in Figure 6B, three out of five fungi, namely PC8, CM10, and CC30, exhibited significantly better results with ethanol-regenerated biomass. Conversely, PN13 and LD36 exhibited higher BPA adsorption following methanol regeneration. In the case of LD36, the difference was negligible and statistically insignificant in the unpaired *t*-test. It is worth noting that the ethanol-regenerated biomass displayed varying sorption capacity retention. The most efficient CC30 fungus, for instance, demonstrated 76% BPA sorption, which corresponded to 94% of its initial efficiency compared to the first batch. The results for the remaining three fungi, LD36, CM10, and PC8, were similarly consistent, with values of 63, 61, and 59%, respectively, representing 83, 76, and 81% of their initial efficiency. In contrast, only one methanol-regenerated PN13 fungus displayed 60% BPA removal, corresponding to 80% of its initial efficiency. These results are similar to those obtained in BPA removal studies by walnut shell-based activated carbon [53]. Consequently, based on our findings and available literature, it is evident that there is no universal regeneration solvent. Lee and colleagues showed that regeneration of porphyrinic porous organic polymer with acetone was possible and the remaining adsorption capacity was high, whereas in our sorbents, acetone was entirely ineffective desorption solvent [43]. In contrast, the findings from our desorption investigations (Figure 6A) indicated that the most effective solvent was methanol. However, Yonten and colleagues reported no BPA desorption from immobilized *P. eryngii* mycelia using methanol. Instead, they found that acetonitrile was the most effective solvent, although the BPA recovery was still 1.5–2.5 times lower than our results obtained with methanol [25]. Ethanol was a second noteworthy solvent for the desorption of BPA in our study, and its efficacy has been previously reported in the scientific literature. Its effectiveness at various concentrations has been documented in studies involving reduced or functionalized graphene oxide or nanocomposites, and the results are consistent with our findings [48,49,54]. Therefore, ethanol may be considered a beneficial regeneration solvent due to its efficiency and less toxicity. Moreover, since different mechanisms are involved in BPA sorption, a mix of solvents is worth considering for even better desorption and prolonging sorbent reusability.

The utilization of biomass that has been tested and proven to effectively sorb BPA and be easily regenerated can serve as an effective sorbent for the elimination of BPA from various aquatic environments. By determining the quantity of BPA absorbed by 25 mg of the sample, the amount of BPA absorbed by 1 g of fungal biomass can be calculated. Moreover, by considering the typical BPA concentration in aquatic systems reported in Europe, the potential volume of treated water can also be estimated. It is important to note that the concentration of BPA varies and depends on factors such as the type of water reservoir, country or region, and level of urbanization [55]. In Table 1, volumes of effluent in wastewater treatment plants, surface waters and drinking water calculated with reference to example BPA concentrations from Europe are summarized.

As can be seen, the possible volume of treated water increases from effluent to drinking water due to decreasing concentrations of BPA in subsequent environments. Although the concentration of BPA in these stages seems minimal, it still poses a threat to disrupt the endocrine system of both aquatic organisms and humans to varying extents [10]. The likelihood of encountering the highest concentration of BPA is greatest for wastewater treatment plants that receive municipal sewage, potential run-off from factories, and landfill leachate. However, this value may vary considerably between WTPs, even differing by an order of magnitude. The use of effluent and sludge as fertilizer, where BPA may also be present due to sorption, subsequently affects the concentrations of BPA in surface waters that often supply drinking water intakes [55,57]. Based on the calculations and considering the concentration of BPA, its elimination from drinking water is promising due to the large volume of water treated with a small amount of sorbent at both the main water intake and on a domestic scale. Furthermore, regarding wastewater treatment, it is worth considering the use of sorbents in domestic sewage treatment plants to eliminate BPA from one of its sources and limit its infiltration into groundwater. However, the elimination of BPA is possible at each of the calculated levels once properly scaled. The application of inactive fungal biomass overcomes the disadvantages of bioremediation processes that involve actively growing mycelium, such as adaptation time of the fungal organism, unfavorable growth conditions, and enzyme catalysis. This is because fungal-based bioremediation primarily focuses on degradation by enzymes secreted by white-rot fungi, and in a minor part, adsorption on the mycelium [58,59]. Inactive biomass is typically distinguished by its greater sorption capacity when compared to active biomass [60]. Additionally, the utilization of fruiting bodies biomass-based sorbents represents a means of employing post-production and post-consumer waste in accordance with the principles of the circular economy.

## 3. Materials and Methods

### 3.1. Biological Material

In the present research, the fruiting bodies of fifty fungi, cultivated (acronyms LE1—TF23) and wild (acronyms SR24—LS50) were put under investigation (Table 2).

The fungi LE1-TF23 were cultivated at Poznań University of Life Sciences, while the fungi SR24-PO49 were collected from natural locations by colleagues of Poznań University of Life Sciences. The LS50 was harvested by the co-workers of the Faculty of Biology and Biotechnology at the Maria Curie-Skłodowska University, Lublin, Poland. Voucher specimens of all fungi used in experiments are deposited in the Faculty of the Biology and Biotechnology at the Maria Curie-Skłodowska University.

### 3.2. Chemicals and Reagents

Methanol, boric acid, citric acid, acetic acid, sodium hydroxide, acetone, hydrochloric acid, phosphoric acid were purchased from Avantor Performance Materials, Gliwice, Poland. Ethanol was purchased from Linegal-Chemicals, Blizne Łaszczyńskiego, Poland. Bisphenol A was purchased from Merck, Darmstadt, Germany.

### 3.3. Biomass Preparation

Fruiting bodies were cultivated using the method previously described [61,62] and then lyophilized. Portions of 5 g of the dried fruiting bodies were subsequently homogenized for 10 s using a blade lab mill (model WŻ-1, ZBPP, Bydgoszcz, Poland) and stored at room temperature until use. A sample of each homogenate was washed four times over the course of 48 h using methanol. The amount of methanol used for the first wash was 10 mL per 1 g of homogenate, while the subsequent three washes used 5 mL/g. Each wash involved stirring the biomass in methanol for 8–12 h, followed by centrifugation for 15 min at 10,000 rpm. After the washing process, the homogenates were vacuum-dried (Concentrator Plus, Eppendorf, Darmstadt, Germany) and stored at room temperature until use.

### 3.4. Fruiting Bodies Screening Test

Samples measuring 25 mg were placed into 2 mL Eppendorf tubes and mixed with 2 mL of BPA solution at a concentration of 100 mg/L (selected based on the calibration curve, R^2^ > 0.99). This solution was prepared from a BPA methanol stock solution at a concentration of 5 mg/mL. The mixture was shaken (800 rpm) at room temperature for 30 min (JWE Electronics WL-972S, Warsaw, Poland). Control samples of homogenate were incubated with distilled water. Afterwards, the samples were centrifuged for 10 min at 13,400 rpm, and the supernatant from each sample was transferred to a 96-well UV-permeable plate and diluted four times. The BPA starter solution was also shaken and centrifuged to check for possible BPA precipitation and deposition during centrifugation. The solution was then transferred to the plate and diluted. Assessed and control samples of supernatants and BPA solution were measured on a microplate reader (Multiskan Go 1510 Microplate Reader, Thermo Fisher Scientific, Ratastie, Finland) at 275 nm. To calculate the rate of BPA removal, the absorbance of the control sample was subtracted from the absorbance of the tested sample, which represented the absorbance of residual BPA present in the supernatant (*S*). This data was then used to calculate the percentage of removed BPA (*R*%) relative to the BPA starter solution (*B*) using the Equation (1). In order to calculate the capacity (*q*) in mg of BPA per g of sorbent, the concentration of BPA in starter solution in mg/L (*C*_0_) was reduced by BPA concentration in the supernatant in mg/L (*C_S_*). The volume of starter solution in L (*V*_0_) and the mass of sorbent in g (*m*) were also included and final results were calculated according to Equation (2).

(1) R%=1−SB×100%

(2) $$q=\frac{(C_{0}-C_{S})\times V_{0}}{m}$$

### 3.5. Incubation Time Influence

In this experiment, samples of 25 mg of selected homogenates were transferred into 2 mL Eppendorf tubes and then mixed with 2 mL of a 100 mg/L BPA solution. Control samples were treated with distilled water. The samples were shaken at room temperature and 800 rpm for a series of time points (5, 15, 30, and 60 min). Three test samples and three control samples were removed from the shaker after each time point and centrifuged. The samples at time 0 were shaken using a vortex (Reax top, Heidolph Instruments, Schwabach, Germany) and then immediately centrifuged, measured, and calculated (Section 3.4).

### 3.6. Temperature of Incubation

Samples of 25 mg of selected homogenates were placed into 2 mL Eppendorf tubes and combined with 2 mL of 100 mg/L BPA solution. Control samples were mixed with distilled water. The temperature tested was 6, 20, and 40 °C. For the 6 and 40 °C temperatures, the BPA solution was precooled or preheated, respectively, before being added to the samples of homogenate. The incubation time varied for each fungal homogenate and was 15 min for PC8, CM10, and PN13, while for the CC30 and LD36 fungi, the time was 30 min at 800 rpm. Starter BPA solution samples were incubated in both time variants to assess the possible BPA precipitation in tested temperatures. After incubation, the samples were centrifuged and measured spectrophotometrically, as described in the Section 3.4.

### 3.7. pH Value of the Incubation Buffer

In this investigation, the solvent utilized for the BPA was the Britton-Robinsons buffer (BR buffer) in the range from 2 to 12, rather than distilled water. The BR buffer was a mixture of 0.04 M boric acid, 0.04 M phosphoric acid and 0.04 M acetic acid titrated with 0.2 M sodium hydroxide to the proper pH value. Samples of 25 mg of homogenates were transferred to 2 mL Eppendorf tubes and mixed with 100 mg/L BPA solution (test samples) or the buffer (control samples), and then shaken. The incubation time was appropriate for each fungal homogenate, as described in Section 3.5. The BPA starter solution was incubated in each buffer and centrifuged to confirm any potential BPA precipitation, especially in buffers with lower pH values.

### 3.8. Operational Stability

Samples of 25 mg of selected homogenates were subjected to a series of experiments in which they were shaken with either 2 mL of a 100 mg/L BPA solution or distilled water (control) in 2 mL Eppendorf tubes at a temperature of 20 °C for an appropriate duration for each homogenate (Section 3.5). After incubation, centrifugation, and absorbance measurement, the maximum BPA removal was calculated (Section 3.4). Subsequently, the supernatant residuals from each sample were removed, and fresh portions of the BPA starter solution were added to the samples, allowing for the BPA removal process to be repeated. This procedure was repeated until the BPA removal efficiency fell within the range of 20 to 25% of the initial efficiency obtained during the first cycle.

### 3.9. BPA Desorption and Carrier Regeneration

Methanol, ethanol, acetone, 10% acetic acid, and water were utilized as solvents for BPA desorption from biomass. Following BPA sorption by the biomass, the supernatant was removed from the sample, and the sediment was poured with 2 mL of each solvent. The samples were shaken for 15 or 30 min, adequate on the outcomes of experiments assessing the impact of time on BPA removal. Afterward, the samples were centrifuged for 10 min at 13,400 rpm, and the supernatant samples were transferred to a 96-well plate, diluted four times with distilled water and measured at 275 nm.

After desorption experiment, the ethanol or methanol supernatant was removed from each sample and remained sorbent was vacuum-dried at 30 °C, for 8 h using a Concentrator Plus (Eppendorf, Hamburg, Germany) and homogenized with manual Wheaton glass homogenizer. Samples were reused for BPA removal according to the description in Section 3.4.

### 3.10. Statistical Analysis and Artwork

For each experiment, at least three independent repeats were conducted, and the results were calculated as the mean of the repeats with ± SD. To determine statistical significance, one-way ANOVA and Tukey’s post-hoc test, Person’s correlation test, as well as an unpaired *t*-test were performed using GraphPad Prism 10.2.2 software (San Diego, CA, USA). The graphical abstract was prepared using BioRender.com.

## 4. Conclusions

The objective of the presented paper was to ascertain the viability of utilizing fungal fruiting bodies as a biomass for the elimination of BPA from aquatic environments. This study also sought to identify the fundamental conditions necessary for this process, assess its potential for reuse, and evaluate the performance of the proposed sorbents in diverse aquatic systems. The findings demonstrated that the proposed sorbents were indeed effective. The biomass of fungal fruiting bodies from 23 cultivated and 27 wild fungal species was found to have varying sorption rates for BPA, with five species exhibiting the highest efficiency. This process was rapid and was thought to be primarily driven by electrostatic interactions. These sorbents were found to be effective over a wide range of temperatures and pH levels, which is advantageous for their potential use in diverse aquatic environments. Although the sorbents’ capacity may decrease over time, they can be easily regenerated using mainly ethanol and, in some cases, methanol, preserving their ability to effectively remove BPA. Given their capacity, fungal fruiting body biomass sorbents have the potential to be utilized for water treatment at various levels, including waste, surface, and drinking water, thereby contributing to improved water quality and waste management. Nevertheless, the study was subjected to certain limitations due to the necessity of using an unnaturally high concentration of BPA for detection; the method was deemed sufficient for the analysis of the species tested. The use of advanced analytical techniques such as HPLC–UV or LC/MS, which require proper sample preparation, would have been time-consuming and costly. However, these methods are feasible and are planned for use in future studies, such as the optimization of mass transfer, the scaling up of the process, and the identification of the optimal system for regeneration and treatment of leachate. Further analysis of the cell wall structure may lead to a better understanding of the interactions between its components and BPA, as well as other emerging contaminants. More advanced analysis i.e., FTIR-ATR, EDS, SEM, BET analysis, will help to characterize the cell-wall as sorbent, its composition, structure, and possible mechanism of adsorption of BPA and other xenobiotics. As such, this kind of research can help fill the gap in knowledge about the sorption process as an independent mechanism for the elimination of micropollutants by higher fungi.

## Figures and Tables

**Table 1:** The amount of BPA (mg) adsorbed on 1 g of fungal sorbent and calculated on this basis volume of effluent wastewater, surface water and drinking water.

Species	BPA Adsorbed in a Single Batch [mg/g]	Calculated Volume of Wastewater Effluent [m^3^/g] ^a^	Calculated Volume of Surface Water [m^3^/g] ^b^	Calculated Volume of Drinking Water [m^3^/g] ^c^
**CM10**	6.56	10.1	18.7	469
**PN13**	6.16	9.48	17.6	440
**PC8**	5.92	9.11	16.9	423
**CC30**	6.00	9.23	17.1	429
**LD36**	5.76	8.86	16.5	411

**^a^**—for the highest measured concentration of 0.65 µg/L in wastewater effluent in Germany [55]. **^b^**—for concentration of 0.35 µg/L in samples of surface waters throughout the Europe [56]. **^c^**—for concentration of 0.014 µg/L in samples of drinking waters throughout the Europe [57].

**Table 2:** Names and acronyms of cultivated and wild fungi used in experiments.

Acronym	Species	Acronym	Species
**Cultivated fungi**			
**LE1**	*Lentinus edodes*	**PN13**	*Pholiota nameko*
**PO2**	*Pleurotus ostreatus*	**HM14**	*Hypsyzigus marmoreus* (white)
**PP3**	*Pleurotus pulmonarius*	**HM15**	*Hypsyzigus marmoreus* (brown)
**POf4**	*Pleurotus ostreatus* var. florida	**HE16**	*Hericium erinaceus*
**PE5**	*Pleurotus eryngii*	**AP17**	*Auricularia polytricha*
**PC6**	*Pleurotus citrinopileatus*	**AAJ18**	*Auricularia auricula-judae*
**PD7**	*Pleurotus djamor*	**AB19**	*Agaricus bisporus* (white)
**PC8**	*Pleurotus columbinus*	**AB20**	*Agaricus bisporus* (brown)
**FV9**	*Flammulina valutipes*	**AA21**	*Agaricus arvensis*
**CM10**	*Clitocybe maxima*	**AB22**	*Agaricus brasiliensis*
**GL11**	*Ganoderma lucidum*	**TF23**	*Tremella fuciformis*
**AC12**	*Agrocybe cylindracea*		
**Wild fungi**			
**SR24**	*Stropharia rugosoannulata*	**GA38**	*Ganoderma applanatum*
**AM25**	*Armillaria mellea*	**GR39**	*Ganoderma resinaceum*
**SC26**	*Sparasis crispa*	**GA40**	*Ganoderma adspersum*
**TV27**	*Trametes versicolor*	**GC41**	*Ganoderma carnosum*
**CC28**	*Coprinus comatus*	**PP42**	*Phellinus pini*
**TE29**	*Tricholoma equestre*	**PR43**	*Phellinus robustus*
**CC30**	*Cantharellus cibarius*	**FF44**	*Fomes fomentarius*
**BE31**	*Boletus edulis*	**FP45**	*Fomitopsis pinicola*
**IB32**	*Imleria badia*	**HA46**	*Heterobasidion annosum*
**LA33**	*Leccinum aurantiacum*	**GO47**	*Gleophyllum odoratum*
**LS34**	*Leccinum scabrum*	**PS48**	*Pholiota squarossa*
**MP35**	*Macrolepiota procera*	**PO49**	*Pleurotus ostreatus*
**LD36**	*Lactarius deliciosus*	**LS50**	*Laetiporus sulphureus*
**PB37**	*Piptoporus betulinus*		

## Data Availability

Data will be made available on request.
